# Susceptibility of *Rhipicephalus microplus* to fluralaner: assessment before and after implementation of a strategic control protocol in taurine cattle in a tropical region

**DOI:** 10.1186/s13071-026-07491-1

**Published:** 2026-06-16

**Authors:** Haile Dean Figueiredo Chagas, Gabriel Webert Gomes, Rafael Assunção Carvalho, Arthur Matos de Santana, Ana Carolinne Lopes Ascenção, Emanuel Magalhães Souza, Bruno César Ferreira Gonzaga, Lorena Lopes Ferreira, Daniel de Castro Rodrigues, Welber Daniel Zanetti Lopes, Caio Marcio Oliveira Monteiro

**Affiliations:** 1 Graduate Program in Biology of the Parasite-Host Relationship, Institute of Tropical Pathology and Public Health, Federal University of Goiás, R. 235, s/n University East Sector, Goiânia, GO CEP 74605‑050 Brazil; 2 Undergraduate Program in Veterinary Medicine, School of Veterinary and Animal Science, Federal University of Goiás, Nova Veneza, km 8, Campus Samambaia, Goiânia, GO CEP 74690‑900 Brazil; 3 Graduate Program in Animal Science, School of Veterinary and Animal Science, Federal University of Goiás, Nova Veneza Highway, Km 8, Campus Samambaia, Goiânia, GO CEP 74690‑900 Brazil; 4 Undergraduate Program in Biotechnology, Institute of Tropical Pathology and Public Health, Federal University of Goiás, University East Sector, R. 235, s/n, Goiânia, GO CEP 74605‑050 Brazil; 5 Faculty of Medicine, Federal University of Goiás, University East Sector, R. 235, s/n, Goiânia, GO CEP 74605‑050 Brazil; 6 Department of Preventive Veterinary Medicine, School of Veterinary Medicine, Federal University of Minas Gerais, Belo Horizonte, MG CEP 31270‑901 Brazil; 7 MSD Animal Health, Avenida Dr. Chucri Zaidan, 246‑96, 9th Floor, São Paulo, SP CEP 04583‑110 Brazil; 8 Department of Biosciences and Technology, Institute of Tropical Pathology and Public Health, Federal University of Goiás, R. 235, s/no, University East Sector, Goiânia, GO CEP 74605‑050 Brazil

**Keywords:** Cattle tick, Isoxazoline, Strategic control, Resistance management

## Abstract

**Background:**

Fluralaner has been used in the strategic control of the *Rhipicephalus microplus* tick, an important ectoparasite for cattle farming. However, the effects of its application on the susceptibility profiles of these tick populations after treatment have not yet been investigated. In this study, we evaluated the effect of a strategic control protocol with fluralaner on the phenotypic susceptibility profile of a population of * R. microplus* before and after its implementation.

**Methods:**

Thirty naturally infested cattle were divided into two experimental groups: T01 (control group): cattle treated with a topical (spray) formulation containing a synthetic pyrethroid and two organophosphates; T02 (strategic control): cattle treated with a formulation (pour-on) containing only fluralaner. For group T01, animals were treated when the average tick count was  ≥30 to allow determination of the infestation challenge on the farm, while animals in group T02 were treated when 30% of the animals had adult ticks < 4 mm. Tick counts on animals were performed every 7 days for 266 days. Larvae monitoring in the pasture was performed every 30 days using a dragging flannel for 240 days. Larval immersion tests (LIT) and adult immersion tests (AIT) were used to assess the phenotypic susceptibility profile of ticks before and after the implementation of the strategic control protocol over a tick season in a tropical region.

**Results:**

The animals in the group treated with fluralaner (T02) had lower mean tick counts (*p* ≤ 0.05) than those observed in the control group (T01) in 35 of the 38 evaluations during the 266 days of the study. In the pasture, the number of larvae in T02 was lower (*p* < 0.05) in seven of the eight evaluations. There was no increase in LC50 values for larvae and engorged females after using the strategic control protocol with fluralaner for 9 months.

**Conclusions:**

The use of the strategic control protocol with fluralaner resulted in a significant reduction in tick numbers on cattle and in pastures, and after 1 year of use, the population remained susceptible to this compound. No upward trend in LC50 values was observed after 9 months of fluralaner use.

**Graphical Abstract:**

**Figure:**
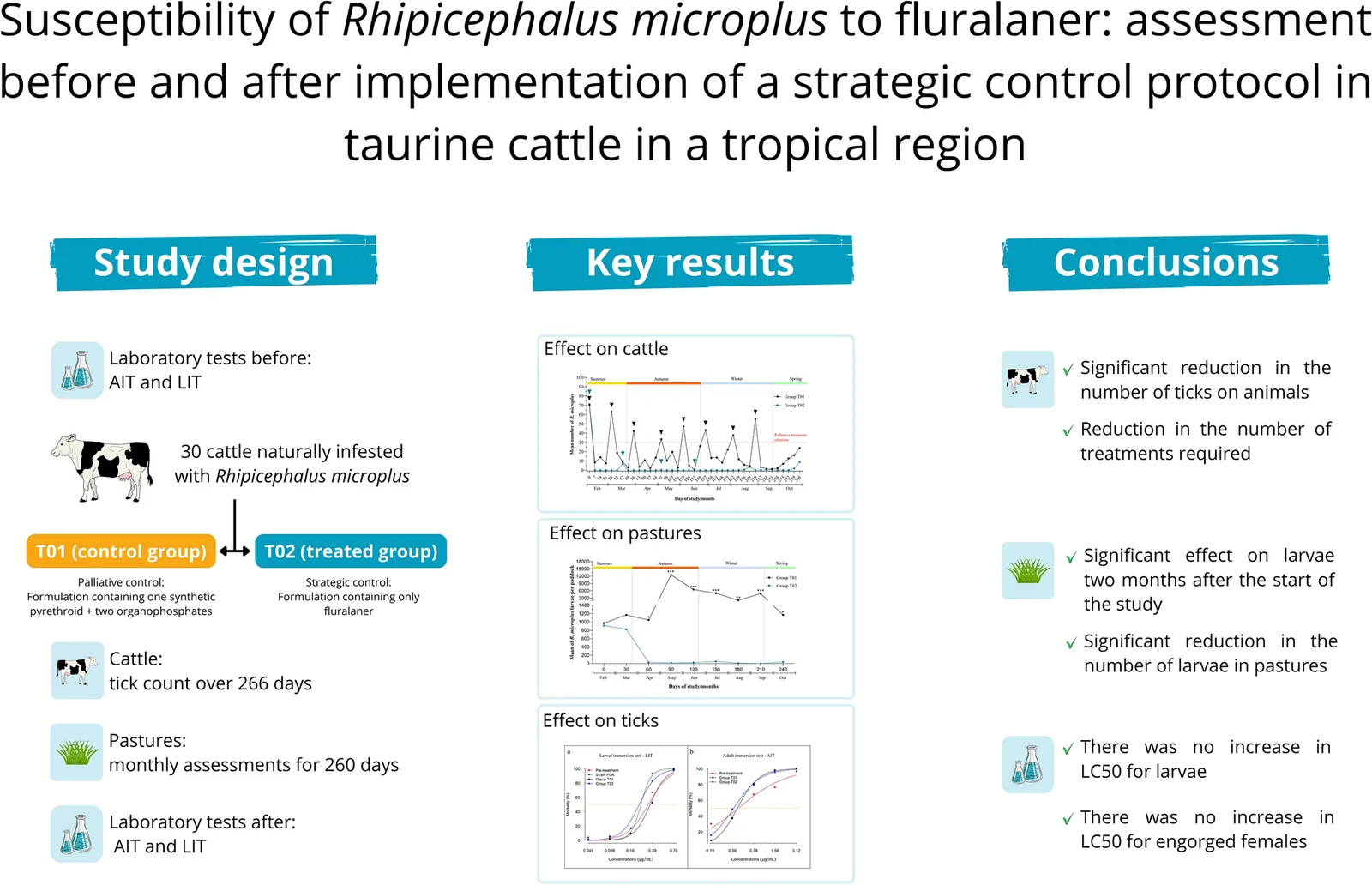

**Supplementary Information:**

The online version contains supplementary material available at 10.1186/s13071-026-07491-1.

## Background

The cattle tick *Rhipicephalus microplus* (Canestrini, 1888) is an important hematophagous ectoparasite that is widely distributed in regions with tropical and subtropical climates [1]. In countries where it occurs, high infestation rates reduce productivity,  facilitate the trasnmission of pathogens (*Anaplasma marginale*, *Babesia bovis*, and *Babesia bigemina*), and generate control costs [2, 3]. In Brazil alone, the economic losses caused by *R. microplus* in cattle farming are estimated at around US$3.2 billion annually [4].

The use of synthetic chemicals acaricides remains the primary tool for controlling this tick [5, 6]. However, the constant use of these products, sometimes without following adequate technical criteria [7], has led to the selection of ticks resistant to almost all chemical classes available on the market, increasing the challenges in controlling *R. microplus* [3, 8, 9]. In this scenario, a new drug (fluralaner) belonging to the isoxazoline class was initially registered in Brazil and then in other Latin American countries for the control of *R. microplus* and other bovine ectoparasites [10, 11].

Strategic control of this ectoparasite consists of applying acaricides at specific times, based on tick population dynamics, to maintain acceptable levels of infestation in pastures and animals, so that this does not affect the health and productive performance of cattle [12, 13]. In addition, one of the objectives of strategic control is to apply as few treatments as possible, thereby reducing selection pressure and delaying the selection of resistant ticks [14, 15]. However, few studies have evaluated the effect of implementing tick control strategies in cattle on the susceptibility profile of *R. microplus* populations [6, 16].

The effect of the strategic tick control protocols with fluralaner on cattle was evaluated across different regions, breeds, and production systems, demonstrating high efficacy, improved animal productivity, and no impact on the establishment of enzootic stability of tick fever agents [13, 17–20]. However, there are still no data regarding the effect of fluralaner treatments on the phenotypic susceptibility of *R. microplus* before and after the implementation of strategic control protocols using this isoxazoline [6, 16].

The susceptibility profile of a tick population can be assessed using laboratory tests, including adult immersion tests (AIT), larval packet tests (LPT), and larval immersion tests (LIT) [2, 3, 21]. Through these bioassays, it is possible to evaluate a dose-response relationship and determine a lethal concentration (LC). These values allow LCs to be compared across different populations and ticks' susceptibility profiles to be determined [2, 16]. These tests can also be performed with discriminating doses (DDs), in which one or two concentrations are tested to assess the susceptibility profile of a population [22, 23]. Using these methodologies, it is possible to analyze the same population before and after the application of different treatment protocols, detecting changes in the phenotypic susceptibility profile over time [6, 16]. In this context, the present study evaluated: (i) the effect of implementing a strategic control protocol using fluralaner to control *R. microplus* in taurine cattle in a tropical climate region; (ii) for the first time to our knowldege, the effect of using this protocol on the phenotypic susceptibility profile of the *R. microplus* population before and after exposure to fluralaner.

## Methods

### Study period and location

The study was conducted between February and October 2024 on a commercial farm located in the municipality of São Miguel do Passa Quatro (latitude: 17° 3′5.43"S; longitude: 48°41′31.80"W), Goiás, Central-West Brazil, with no history of fluralaner use. The region is part of the Cerrado (Brazilian Savannah) biome and has a tropical climate, with an average annual temperature ranging from 17 °C to 30 °C, with heavy rains from October to April and a drier period from May to September. According to the Köppen-Geiger classification, the area has an “Aw” climate [24]. For this region, three generational peaks of *R. microplus* are expected during the rainy season and two during the dry season, with five generational peaks occurring throughout the year [6, 25].

### Ethical aspects

The study followed the guidelines of Ordinance 48 of the Brazilian Ministry of Agriculture and  Livestock (MAPA) [26] and the World Association for the Advancement of Veterinary Parasitology (WAAAP) [27]. In addition, all recommendations in the reference guides on good practices and animal welfare management were followed, and the study protocol was approved by the Ethics Committee for the Use of Animals (CEUA) of the Federal University of Goiás (UFG) (protocol: 072.2018).

### Acaricides used in the study

In this study, a pour-on acaricide containing only fluralaner as the active ingredient (Exzolt 5%^®^, 1 mL/20 kg, MSD Animal Health) and a spray acaricide containing a combination of a synthetic pyrethroid and two organophosphates (Potenty^®^, concentration of active ingredients in the application mixture: 125 ppm alphacypermethrin + 400 ppm ethion + 212 ppm chlorpyrifos; 12.5 mL of product/5Ll of spray/animal, MSD Animal Health) were used. The acaricides were provided by MSD Animal Health.

### Formation and randomization of groups for strategic control

The strategic control study was initiated on February 2, 2024 (D0) to allow the animals to reach a sufficient level of tick burden from the first generation. For that, the animals were visually inspected once a week until a sufficient level of infestation was observed (approximately 100 females per animal: 4.5–8.0 mm in length). This was necessary to obtain the required number of *R. microplus* engorged females for laboratory testing before the strategic control protocol could be implemented.

**Fig. 1:**
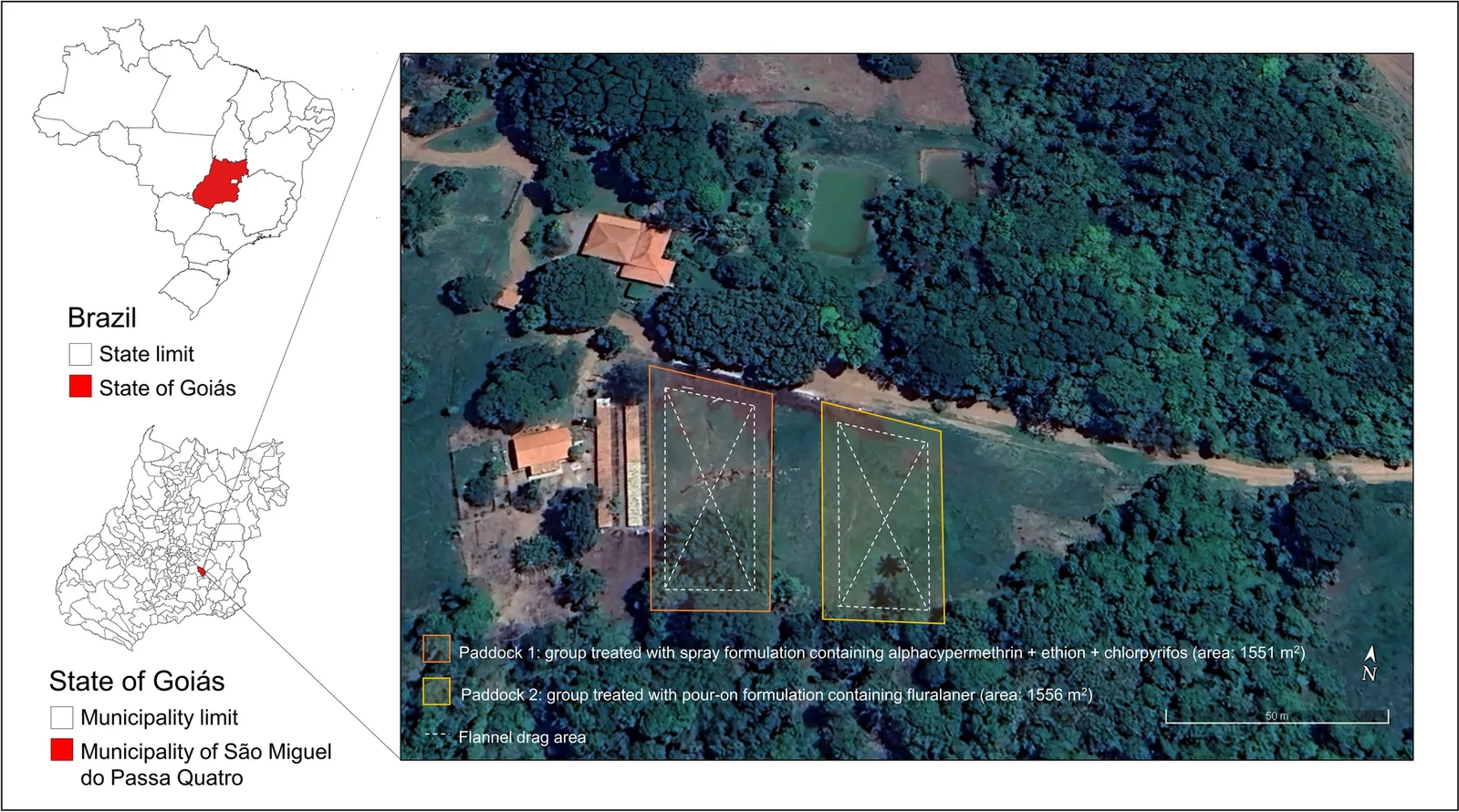
Location of the study area where the animals in the control group (T01 - paddock 1) treated with the spray formulation (cypermethrin, chlorpyrifos, and ethion) and the animals in the group treated with the fluralaner formulation (T02 - paddock 2) were kept

To conduct the study, 30 Holstein heifers, with an average age of 10 months and naturally infested with *R. microplus*, were selected. Only healthy animals with good nutritional status and body condition scores were included in the study. The last acaricide treatment was performed 90 days before the start of the study with the commercial formulation containing two organophosphates and a synthetic pyrethroid (Potenty^®^). The animals used in the study had been allocated to these paddocks 3 months before the experiment began. Since the number of cattle, age, breed, and management conditions were similar between groups, this resulted in comparable levels of pasture infestation by tick larvae (*p *> 0.05). Before the start of the study, female ticks measuring 4.5 to 8.0 mm were counted on all animals, and some cattle were redistributed to ensure greater homogeneity between groups regarding infestation levels (*p* > 0.05). The formation of the blocks was based on the arithmetic mean number of females measuring between 4.5 and 8.0 mm on one side of the cattle (always counted on the same side), without multiplying the counts by two, obtained from counts performed on days − 3, − 2, and − 1, as recommended by Wharton & Utech [28]. The animals were distributed into two blocks containing 15 cattle each, which were randomly allocated to one of the groups: T01 (control) and T02 (fluralaner). Each group of 15 animals was kept in a paddock of approximately 1550 m^2^ containing Coast cross grass (*Cynodon dactylon*) (Fig. 1). During the study, the cattle received water and mineral supplementation *ad libitum* and a mixture of silage and feed twice a day.

### Treatment protocols and counting of *R. microplus* on animals

To conduct this study, the strategic control protocol for taurine cattle in tropical areas was adopted, using fluralaner, in accordance with criteria established by Aquino et al. [13]. The counts of female *R. microplus* (4.5–8.0 mm) on each animal were performed weekly by the same person, at the same time, and on the same side of the animal, without multiplying the count by two, according to Wharton & Utech [28]. These counts were performed on days + 7, + 14, + 21 and every 7 days until day + 266. This tick population shows resistance to synthetic pyrethroids, formamidines, organophosphates, phenylpyrazoles, and macrocyclic lactones (unpublished data), and susceptibility to isoxazolines [23].

The cattle in group T01 (control group) were treated with a spray formulation containing a combination of a synthetic pyrethroid and two organophosphates (Potenty^®^) when the mean tick count for the group was ≥ 30, according to criteria pre-established by Gomes et al. [12]. It is important to note that the adopted treatment protocol does not interfere with the life cycle of *R. microplus*, since females ≥ 4 mm undergo rapid engorgement and detach from the host within 24 h [28]. Furthermore, the chosen product has no residual effect against new infestations of *R. microplus* and thus does not alter the population dynamics of this tick [29]. Thus, it was possible to observe the challenge regarding tick infestation on the farm where the study was conducted. The acaricide used in group T01 was selected based on the results of the adult immersion test (AIT), showing > 95% efficacy (unpublished data).

In group T02, the animals were treated with fluralaner (Exzolt 5%^®^) on day 0; however, for retreatment, the methodology adopted by Nicaretta et al. [29] was used. In this methodology, the animals were treated again with fluralaner when at least 30% (5/15) of the animals in this group had ticks < 4 mm attached between their hind legs and dewlap (the preferred attachment site for *R. microplus*). Before each treatment, the animals were weighed to calculate the volume of acaricide to be administered, in accordance with the manufacturer's recommendations.

### Counts of *R. microplus* larvae in pastures

In the pasture, the number of larvae was assessed using the flannel drag technique. The dragging was performed using cotton flannel (75 × 100 cm) over the ground-level vegetation, according to the methodology used by Nicaretta et al. [29]. Three flannels were used per paddock; each flannel was dragged for 300 linear meters, that is, the drag occurred in 900 linear meters for each paddock (Fig. 1). After dragging, the flannels were placed in properly identified plastic bags and subsequently sealed. The flannels were stored in a freezer (− 20 °C) until the number of larvae was quantified using a clinical aspirator (Aspiramax, MA-520, Omron). The counted larvae were transferred to bottles containing 70% ethanol, and a portion of them were identified to genus level, according to Clifford & Anastos [30]. Based on this identification, the tick species present in the pastures were extrapolated. The dragging was performed on days 0, 30, and every 30 days until day 240, always occurring in the first half of each month.

### Assessment of the susceptibility profile before and after implementation of treatment protocols

To assess the susceptibility profile of ticks to acaricides, tests were performed with unfed larvae and engorged females from samples (engorged females) collected before the start of strategic control implementation and at the end of the study (T01 and T02). The ticks obtained after the T01 and T02 treatment protocols were named subpopulation 1 and subpopulation 2, respectively. Some of the females collected were used to perform the Adult Immersion Test (AIT) on engorged females, while others were incubated in a biological oxygen demand (BOD) incubator (SL200, Solab) at 27 ± 1 °C and 80 ± 5% RH for oviposition. The collected egg masses were weighed in 250-mg aliquots and incubated under the same conditions to obtain larvae for the larval immersion test (LIT). For laboratory assays, engorged females recently detached from the animals and larvae with 15 to 21 days old after hatching were used.

At the LIT, a reference strain (Porto Alegre, POA) susceptible to acaricides was used for comparison with the data obtained in tests using ticks collected before and after the implementation of the strategic control protocol with fluralaner.

### Larval immersion test (LIT) to determine the lethal concentration (LC50) with fluralaner

The LIT was performed as recommended by Rodrigues et al. [11]. For this test, the commercial product Exzolt 5%^®^ was used as the source of fluralaner. Dimethyl sulfoxide at 2% (DMSO) was used as the solvent because of its low toxicity to *R. microplus* larvae [31].

In the LIT, approximately 500 unfed larvae were transferred to a plastic microtube (1.5 mL) containing the test solution, which was vigorously shaken for 3 min. The solution was then dispensed, and approximately 100 larvae were added to the center of a filter paper (6 cm × 6 cm) that had been previously folded in half and sealed at the ends with paper clips. Concentrations of 0.024, 0.048, 0.095, 0.19, 0.39, 0.78, 1.56, and 3.125 μg/mL were tested. Two control groups were formed, one with distilled water and the other with 2% DMSO. For each group, five replicates were used, and the assays were performed in duplicate. The packages were placed in a BOD incubator (27 ± 1 °C and RH 80 ± 5%) for 24 h, after which the mortality rate was assessed. Larvae that did not move after stimulation with CO_2_ were considered dead.

### Larval immersion test (LIT) with discriminating doses (DDs) of fluralaner

The LIT with DDs was performed as described above. The DDs tested (1.55 and 3.12 μg/mL) were proposed by Rodrigues et al. [11]. In addition, two control groups were formed, one with distilled water and the other with the solvent (DMSO 2%). For each group, 10 replicates were used, and the assays were performed in duplicate.

### Adult immersion test (AIT) with fluralaner for determining lethal concentration (LC50)

The AIT was performed according to Drummond et al. [32], with adaptations according to Silva [33]. The engorged females were divided into groups of 10 ticks with previously homogenized weights (*p* = 0.99) and were immersed for 3 min in solutions containing different concentrations of fluralaner (0.19, 0.39, 0.78, 1.56, 3.12, 6.25, and 12.5 μg/mL). Two control groups were also formed, as mentioned for the LIT. In the studies, 10 replicates were used, and the assays were performed in duplicate.

After immersion, the females were removed from the solutions, placed on paper towels to remove excess solution, and then placed in 12-well cell culture plates (one female/experimental unit per well, one plate per treatment) for reproductive biology assessment. The ticks were placed in a BOD incubator with controlled temperature and humidity (27 ± 1 °C and RH 80 ± 5%) for evaluation of biological parameters. After 15 days, the eggs were weighed, placed in plastic syringes with the distal ends cut off, sealed with cotton wool, and incubated under the same temperature and humidity conditions. After 21 days, the hatching percentage was evaluated by quantifying larvae and eggs by sampling [34].

The following biological parameters were evaluated: female weight before oviposition (FWBO, mg), egg mass weight (EMW, mg), and larval hatch percentage (LHP, %). These values were used to calculate the egg production index (EPI, %) [35], oviposition inhibition index (OII, %) [31], and percent control (C, %) [32], using the following formulas:

- Egg production index $\left( {EPI} \right)\, = \,\frac{EMW}{{FWBO}}\, \times \,100$
- Oviposition inhibition index:
  o Oviposition index $$\left( {OI} \right)\, = \frac{EMW}{{FWBO}}$$
  o Oviposition inhibition index $\left( {OII} \right)\, = \,\frac{OI\,\,of\,the\,treated\,group}{{OI\,of\,the\,contrl\,group\,}}\, \times \,100$
- Estimated reproduction $$\left( {ER} \right)\, = \,\left( {\frac{EMW}{{FWBO}}} \right)\,\, \times \,\,LHP\, \times \,\,20.000$$
- Percent control $$\left( {CP} \right)\, = \,\left( {\frac{ER\,of\,the\,control\,group\, - \,ER\,of\,the\,treated\,group}{{ER\,of\,the\,control\,group\,}}} \right)\, \times \,100$$

**Statistical Analysisbr** The analyses were performed using R-Studio software (RStudio 2024.12.1 + 563 – Posit Software). The number of ticks counted on the animals was not normally distributed, so the nonparametric Kruskal-Wallis test was used. The data obtained from the larval count on the flannel cloths in each paddock showed a normal distribution, so analysis of variance (ANOVA) was used. The values of the biological parameters obtained through the AIT were tested for data normality using the Shapiro-Wilk test. Parametric data were analyzed using analysis of variance (ANOVA) followed by Tukey’s test (*p *< 0.05), while nonparametric data were analyzed using the Kruskal-Wallis test followed by Dunn's test (*p* < 0.05), with Bonferroni adjustment. The lethal concentration 50 (LC50) was calculated from the larval mortality for LIT and from the percentage of efficacy (percentage of control) for AIT, using Probit analysis [36].

The resistance ratio for larvae was calculated in two ways: RR1: resistance ratio obtained from the LC50 value of the field strain (before or after treatment), divided by the LC50 value of the POA strain [2]; RR2: resistance ratio obtained from the LC50 value after treatment (T01 = subpopulation 1 or T02 = subpopulation 2), divided by the LC50 value of the field population before treatment [37]. For engorged females, only the second calculation proposal was performed. The population was considered susceptible when the RR was ≤ 3 [11]. For DD tests, samples with mortality > 95% were considered susceptible to fluralaner [38].

## Results

### Tick counts and efficacy of treatment protocols on cattle

**Fig. 2:**
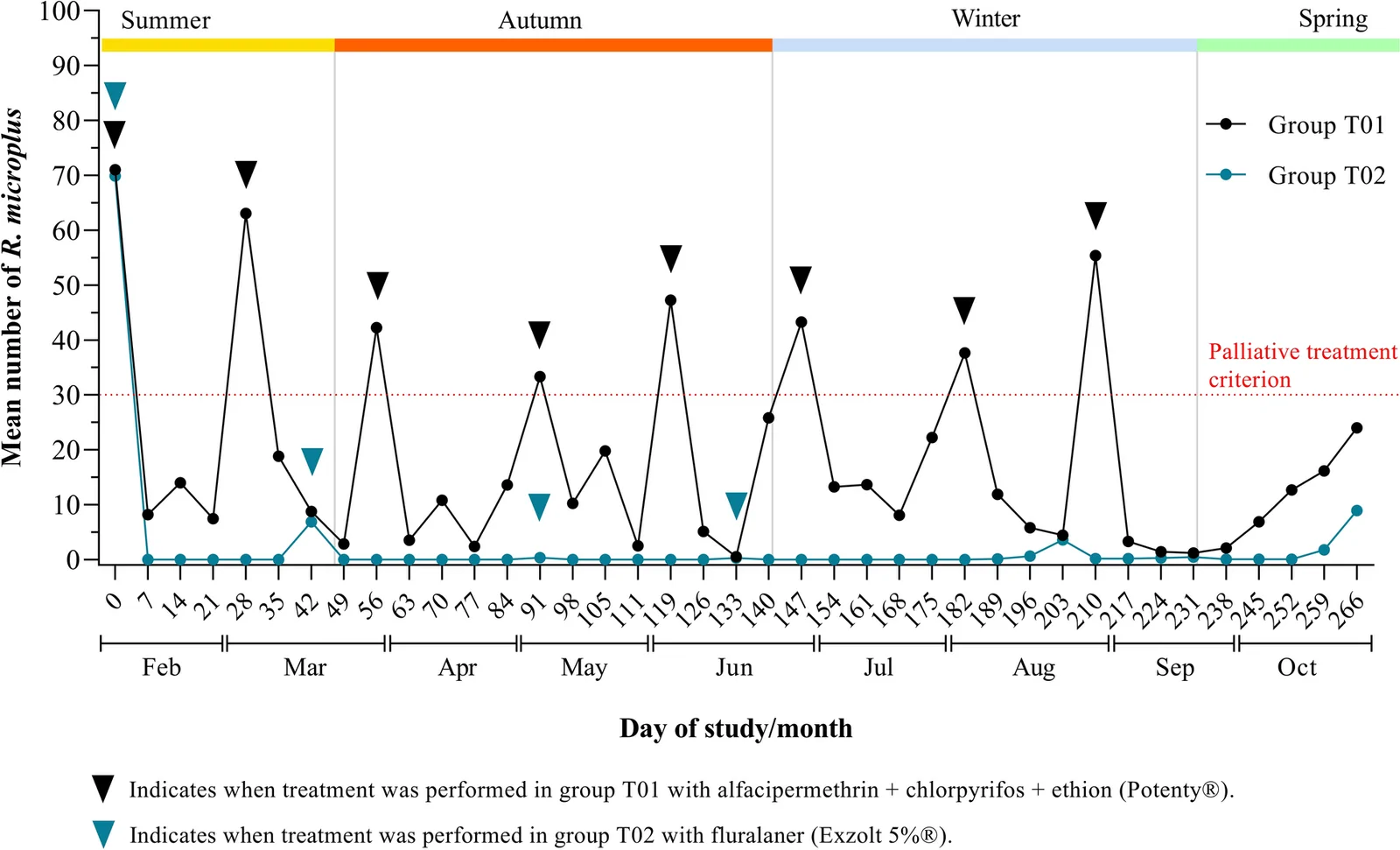
Mean number Rhipicephalus microplus females (between 4.5 and 8 mm in length) counted on the left side of cattle treated with the spray formulation containing alphacypermethrin + chlorpyrifos + ethion (Potenty®, Group T01) or with the pour-on formulation of fluralaner (Exzolt 5%®, Group T02), during 266 days

On day 0, animals in both groups had a mean of approximately 70 ticks, and no difference was observed in the mean tick count between animals in groups T01 and T02 (*p* = 0.718). In group T01 (control group), after day 0, the mean tick count was > 30 on 7 days of evaluation. From day 0 to day 266 (~ 9 months), the animals from the group T01 received eight spray treatments (0, + 28, + 56, + 91, + 119, + 147, + 182, and + 210), corresponding to two treatments during the summer, three in the autumn, and three in the winter (Fig. 2). The intervals between treatments for this group ranged from 28 to 35 days, according to the established criteria; however, after the treatment on day + 210, at the end of winter, there was no need for further treatment (Fig. 2; Additional file 1: Table S1).

For group T02 (fluralaner), starting with the first treatment with the fluralaner pour-on formulation, the tick count was very close to 0 at almost all evaluation points, differing (*p* < 0.05) from the control group (T01) in 35 of the 38 evaluations, except for the counts on days + 133, + 203, and + 231, where the number of ticks was similar between groups (*p *> 0.05). Due to the retreatment criteria established for this group, four treatments were necessary throughout the study period. The animals were treated on days 0, + 42, + 91, and + 133, corresponding to two treatments in the summer and two in the autumn, with intervals of 42 to 49 days between treatments. After day + 133, at the end of autumn, no new treatment was necessary until the end of the study in this group (Fig. 2; Additional file 1: Table S1).

During the study period, no adverse effects were observed in the animals after administration of both commercial acaricides. Furthermore, no other acaricide treatment was administered concomitantly with the treatments planned for each group.

### Larval infestation in pasture

**Fig. 3:**
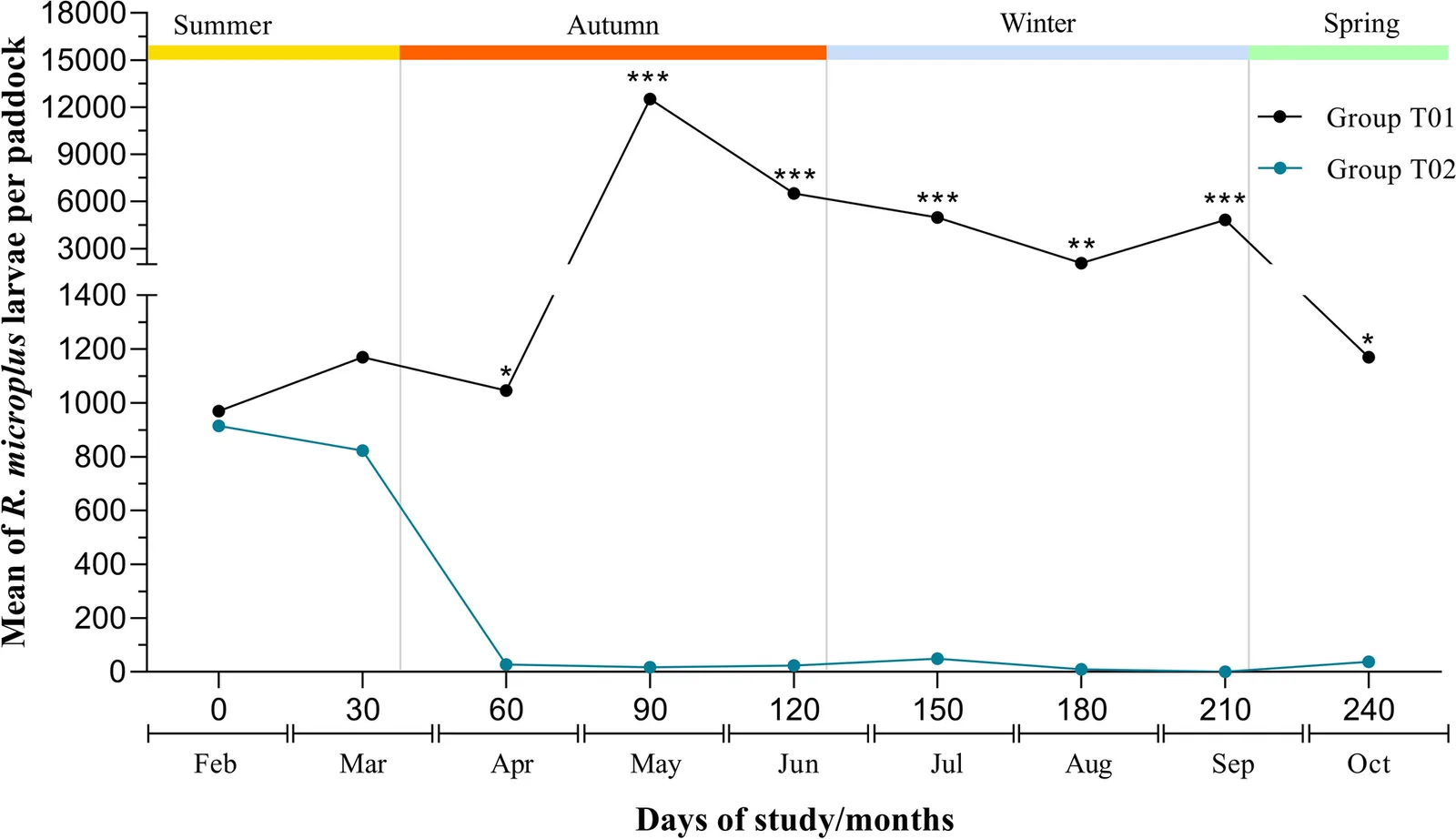
Mean number of Rhipicephalus microplus larvae collected by dragging flannel cloths in the paddocks where animals treated with a spray formulation containing alphacypermethrin + chlorpyrifos + ethion (Potenty®, Group T01) or a pour-on formulation of fluralaner (Exzolt® 5%, Group T02) were kept over a 9-month period. Significance levels are indicated as follows: p < 0.05; p < 0.01; p < 0.001

The larvae collected from the paddocks using the flannel drag technique, where animals from groups T01 and T02 were maintained, were identified to the genus level as *Rhipicephalus*. For the paddock where the animals from group T01 (control group) remained, on day 0 (summer), the mean number of larvae was 969 (average number obtained from the three cloth samples). Higher numbers (> 1000) were observed in the following months. The mean number of larvae counted on days + 30 (summer), + 60, + 90, and + 120 (autumn) was 1169.3, 1046.3, 12,516.0, and 6513.7, respectively. In the evaluations on days + 150, + 180, and + 210 (winter), the mean number of larvae counted was 4978.3, 2075.7, and 4839.7, respectively. During spring, on day + 240, the number of larvae was 1169.7 (Fig. 3).

In the paddock where the animals from group T02 (fluralaner) remained, there was no difference in the mean number of larvae recovered on days 0 (*p* = 0.341) and + 30 (*p* = 0.327) compared with the control group (Fig. 3). However, 60 days after the first treatment with fluralaner, the mean number of larvae was reduced to 27.3, differing significantly from the number of larvae observed in the control group (*p *= 0.033). At all other evaluation times (+ 90, + 120, + 150, + 180, + 210, and + 240), the mean number of larvae was ≤ 50, with significant differences (*p* < 0.01) compared with the number of larvae observed in the control group, in which the mean was always > 1000 larvae. It should be noted that the last treatment occurred on day + 133, at the end of autumn, and even so, the number of larvae remained low (< 49.3) during winter and early spring (Fig. 3).

### Lethal concentration (LC50) and resistance ratio (RR) of fluralaner

**Fig. 4:**
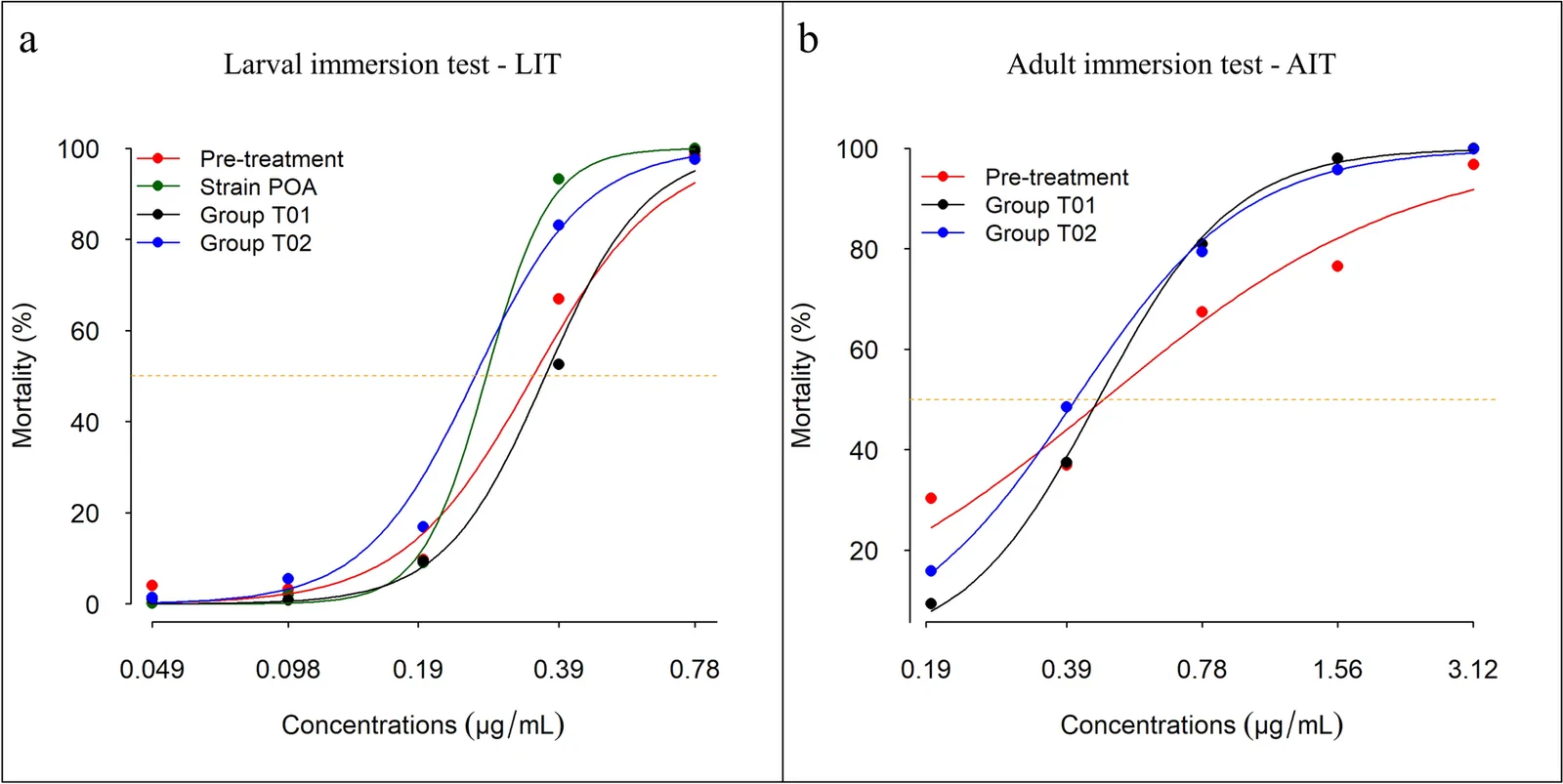
Dose-response curves of unfed larvae (a) and engorged females (b) of Rhipicephalus microplus exposed to different concentrations of fluralaner. Lines and dots indicate: green – POA strain; red – field population before treatment; black – Group T01 after treatment with alphacypermethrin + chlorpyrifos + ethion (Potenty®); blue – Group T02 after treatment with the pour-on formulation of fluralaner (Exzolt 5%®); orange dotted line – lethal concentration capable of killing 50% of ticks (LC50)

In the LIT, with different concentrations of fluralaner, before the implementation of treatment protocols (pre-treatment), an LC_50_ of 0.35 (95% CI 0.35–0.36) μg/mL was observed for unfed larvae. After 266 days of field study, for group T01 (subpopulation 1, control), the LC50 was 0.36 (95% CI 0.35–0.37) μg/mL, and for group T02 (subpopulation 2, treated only with fluralaner), the LC50 was 0.25 (95% CI 0.25–0.26) μg/mL. For the POA strain, the LC_50_ was 0.32 (95% CI 0.32–0.33) μg/mL. Considering the RR calculation using the POA strain as reference, values of 1.09, 1.13, and 0.78 were observed for field ticks obtained before the division of the groups (pre-treatment), after treatment alphacypermethrin + chlorpyrifos + ethion (control group T01), and after treatment with fluralaner (group T02), respectively. In the RR calculation, using the field strain before exposure to the treatments as reference (pre-treatment), RR values of 1.03 and 0.71 were observed for ticks from groups T01 and T02, respectively. In both cases, the RRs obtained were ≤ 3, allowing: (i) classification of the field population as susceptible to the POA strain; (ii) observation that ticks after acaricide treatments (T01 and T02) remained susceptible to fluralaner during the study (~ 9 months) (Table 1; Fig. 4a). Furthermore, no trend toward increasing LC50 values was observed.

**Table 1:** Lethal concentrations (LCs) and resistance ratios (RRs) for unfed larvae of *Rhipicephalus microplus* exposed to different concentrations of fluralaner under laboratory conditions (27 ± 1 °C and RH of 80 ± 10%) before and after the implementation of strategic control protocols

Period/group	LC50 μg/mL	95% CI	Slope	RR^1^	Status	RR^2^	Status
POA	0.32	0.32 – 0.33	6.63	…	…	…	…
Pre-treatment*	0.35	0.35 – 0.36	3.19	1.09	Susceptible	…	…
Group T01	0.36	0.35 – 0.37	3.87	1.13	Susceptible	1.03	Susceptible
Group T02	0.25	0.25 – 0.26	3.58	0.78	Susceptible	0.71	Susceptible

POA = test with the Porto Alegre strain, susceptible to acaricides

95% CI = 95% confidence interval

RR^1^= resistance ratio obtained from the LC_50_ value of the field strain (before or after treatment), divided by the LC50 of the POA strain

RR^2^ = resistance ratio obtained from the LC_50_ value after treatment (T01 or T02), divided by the LC_50_ of the field population before treatment

^*^Initial test performed before the division of the groups to carry out strategic control study

Group T01: animals treated with the formulation containing alphacypermethrin + chlorpyrifos + ethion (Potenty^®^). Laboratory tests performed after completion of the strategic control study

Group T02: animals treated with the formulation containing fluralaner (Exzolt 5%^®^). Laboratory tests performed after completion of the strategic control study

### Larval immersion test (LIT) with discriminating doses (DDs) of fluralaner

For LIT with fluralaner, regardless of the DD tested, 100% mortality was observed in ticks obtained from both treated groups before and after the treatment protocols, and the same was observed in tests with the POA strain (Table 2). Thus, through this analysis, it was also possible to observe that the two subpopulations remained susceptible to fluralaner after the study period (266 days) and implementation of the protocols with four treatments with fluralaner.

**Table 2:** Mortality percentage of unfed larvae of *Rhipicephalus microplus* before and after strategic control protocols, exposed to two discriminating doses (DD) of fluralaner, under laboratory conditions (27 ± 1 °C and RH of 80 ± 10%)

Period/group	Mortality (%)		
	Control (DMSO 2%)	DD: 1.55 µg/mL	DD: 3.16 µg/mL
POA	1.0 ± 1.3 a	100.0 ± 0.0 b	100.0 ± 0.0 b
Pre-treatment*	1.9 ± 1.3 a	100.0 ± 0.0 b	100.0 ± 0.0 b
Group T01	1.1 ± 1.2 a	100.0 ± 0.0 b	100.0 ± 0.0 b
Group T02	0.7 ± 1.3 a	100.0 ± 0.0 b	100.0 ± 0.0 b

Means followed by different letters in the same row are significantly different at the 5% level

POA = test with the Porto Alegre strain, susceptible to acaricides

DMSO 2% = dimethyl sulfoxide

^*^Initial test performed before the division of the groups to carry out strategic control study

Group T01: animals treated with the formulation containing alphacypermethrin + chlorpyrifos + ethion (Potenty^®^). Tests performed after the end of the study

Group T02: animals treated with the formulation containing fluralaner (Exzolt 5%^®^). Tests performed after the end of the study

### Adult immersion test (AIT) with fluralaner to determine the lethal concentration (LC50) and resistance ratio (RR)

In the AIT, engorged females from all groups treated with distilled water and DMSO 2% had egg production index and larval hatching rates > 45 and 85%, respectively, indicating that the ticks were suitable for testing. In none of the three groups were significant differences (*p* = 0.99) observed for the mean weight of engorged females before oviposition (Table 3). These results indicate that the changes observed in the other biological parameters are effects of the treatments.

**Table 3:** Average weight of females before oviposition, egg mass weight, egg production index, larval hatch, oviposition inhibition index, and percent control of engorged *Rhipicephalus microplus* females treated with fluralaner under laboratory conditions (27 ± 1 °C and RH 80 ± 5%) before and after implementation of the treatment protocols

Test group	Treatments	Female weight before oviposition (mg)	Egg mass weight (mg)	Egg production index (%)	Larval hatch (%)	Oviposition inhibition index (%)	Percent control (%)
Pre-treatment	Distilled water	205.6 ± 55.0 a	102.8 ± 34.1 a	49.7 ± 6.4 a	93.5 ± 7.3 a	-	-
	DMSO 2%	201.7 ± 44.5 a	98.1 ± 25.6 a	49.3 ± 6.2 a	84.4 ± 14.7 a	0.0	12.3
	0.19 μg/mL	204.1 ± 57.5 a	88.0 ± 54.5 a	40.3 ± 25.6 ab	75.6 ± 27.4 a	17.1	30.3
	0.39 μg/mL	204.0 ± 51.3 a	77.0 ± 51.6 ab	35.1 ± 19.7 b	78.3 ± 25.3 ab	28.3	36.9
	0.78 μg/mL	210.1 ± 50.2 a	58.3 ± 53.9 b	25.3 ± 21.3 c	55.0 ± 40.8 c	48.1	67.4
	1.56 μg/mL	201.0 ± 47.4 a	31.5 ± 51.0 c	13.5 ± 20.6 d	50.0 ± 48.2 bc	76.5	83.2
	3.12 μg/mL	202.5 ± 49.1 a	4.8 ± 22.6 d	2.2 ± 10.3 e	24.1 ± 48.2 c	96.8	95.1
	6.25 μg/mL	202.8 ± 46.4 a	0.0 ± 0.0 d	0.0 ± 0.0 e	-	100.0	100.0
	12.5 μg/mL	206.2 ± 50.4 a	0.0 ± 0.0 d	0.0 ± 0.0 e	-	100.0	100.0
T01	Distilled water	248.9 ± 35.0 a	119.6 ± 27.2 a	47.9 ± 9.1 a	87.8 ± 9.8 a	-	-
	DMSO 2%	249.4 ± 33.4 a	122.6 ± 25.4 a	49.1 ± 7.0 a	86.4 ± 17.6 a	0	0
	0.19 μg/mL	248.9 ± 36.0 a	120.8 ± 25.8 a	48.3 ± 7.1 a	78.9 ± 23.8 a	0	9.3
	0.39 μg/mL	249.4 ± 33.3 a	100.2 ± 33.4 b	39.9 ± 13.3 b	65.7 ± 35.5 a	16.4	37.4
	0.78 μg/mL	248.5 ± 33.6 a	31.2 ± 51.6 c	11.8 ± 19.5 c	64.1 ± 43.2 a	73.9	80.9
	1.56 μg/mL	248.4 ± 35.6 a	3.9 ± 13.2 d	2.2 ± 8.0 d	53.7 ± 42.0 a	96.7	98.0
	3.12 μg/mL	248.2 ± 35.0 a	0.0 ± 0.0 d	0.0 ± 0.0 d	-	100.0	100.0
	6.25 μg/mL	250.0 ± 38.7 a	0.0 ± 0.0 d	0.0 ± 0.0 d	-	100.0	100.0
	12.5 μg/mL	248.6 ± 34.1 a	0.0 ± 0.0 d	0.0 ± 0.0 d	-	100.0	100.0
T02	Distilled water	241.0 ± 38.7 a	131.8 ± 25.5 a	54.5 ± 4.8 a	88.2 ± 14.0 a	-	-
	DMSO 2%	238.8 ± 36.3 a	136.1 ± 20.5 a	57.1 ± 4.0 a	86.8 ± 13.5 a	0	0
	0.19 μg/mL	241.5 ± 36.2 a	126.4 ± 34.2 a	51.8 ± 13.0 a	77.5 ± 25.9 a	4.3	15.9
	0.39 μg/mL	240.9 ± 34.9 a	117.1 ± 24.8 a	48.9 ± 9.0 a	51.1 ± 25.9 b	11.1	48.5
	0.78 μg/mL	251.7 ± 27.4 a	68.6 ± 66.0 b	25.4 ± 23.9 b	36.4 ± 33.5 b	50.2	79.4
	1.56 μg/mL	243.5 ± 35.9 a	11.3 ± 30.5 c	4.4 ± 11.9 c	44.6 ± 35.8 b	91.6	95.7
	3.12 μg/mL	242.0 ± 36.9 a	0.3 ± 1.1 c	0.1 ± 0.4 c	56.0 *	99.8	99.9
	6.25 μg/mL	244.7 ± 36.6 a	0.0 ± 0.0 c	0.0 ± 0.0 c	-	100.0	100.0
	12.5 μg/mL	239.7 ± 36.1 a	0.0 ± 0.0 c	0.0 ± 0.0 c	-	100.0	100.0

Averages followed by different letters in the column indicate significant differences (*p* < 0.05) within the same test group

^*^Statistical analysis not performed because of the small sample size, as there was only one experimental unit (1 female) for this treatment

Pre-treatment: initial test performed before the division of the groups to carry out strategic control study

Group T01: animals treated with the formulation containing alphacypermethrin + chlorpyrifos + ethion (Potenty^®^). Tests performed after the end of the study

Group T02: animals treated with the formulation containing fluralaner (Exzolt 5%^®^). Tests performed after the end of the study

In the experiment with engorged females recovered before the implementation of the strategic control protocol (pre-treatment), a reduction (*p* < 0.01) in the egg mass weight was observed from the concentration of 0.78 μg/mL, and the same was observed regarding the larval hatch percentage. No egg masses, and consequently larval hatch, were detected from engorged females treated with the concentrations of 6.25 and 12.5 μg/mL, and these results were statistically equal to the 3.12 μg/mL concentration. The oviposition inhibition index and percent control were > 95% at the three highest concentrations of fluralaner tested (3.12, 6.25, and 12.5 μg/mL).

In the study with subpopulation 1 (T01), the same was observed for the engorged female oviposition, with a significant reduction (*p* < 0.01) of the egg mass weight from the concentration of 0.78 μg/mL; however, for larval hatch, no significant reduction was observed until the concentration of 1.56 μg/mL. No egg masses, and consequently larval hatch, were detected from engorged females treated with the concentrations of 3.12 at 12.5 μg/mL, and these results were statistically equal to the 1.56 μg/mL concentration. The oviposition inhibition index and percent control were > 95% at the four highest fluralaner concentrations (1.56, 3.12, 6.25, and 12.5 μg/mL).

In the study with subpopulation 2 (T02), a reduction (*p* < 0.05) in the engorged females was observed starting from a concentration of 0.78 μg/mL, and a reduction in larval hatch (*p* < 0.05) was observed starting from a concentration of 0.39 μg/mL. No egg masses, and consequently no larval hatch, were detected from engorged females treated with the concentrations of 6.25 and 12.5 μg/mL, and these results were statistically equal to the 1.56 μg/mL and 3.12 μg/mL concentrations. The oviposition inhibition index was > 95% at the three highest concentrations (3.12, 6.25, and 12.5 μg/mL), and the percent control was > 95% at the four highest concentrations (1.56, 3.12, 6.25, and 12.5 μg/mL) (Table 3).

Based on the percentage of efficacy/control obtained in the AIT for ticks collected before the study (pre-treatment), the LC_50_ was 0.47 μg/ml (95% CI 0.45–0.49). After 266 days of study, the LC50 for engorged females recovered from groups T01 and T02 was 0.46 μg/mL (95% CI 0.44–0.47) and 0.41 μg/mL (95% CI 0.39–0.42), respectively. The RR was 0.98 and 0.87 for subpopulations 1 (T01) and 2 (T02), respectively (Table 4; Fig. 4b). So, two subpopulations remained susceptible to fluralaner after the study period (266 days) and implementation of the treatment protocols (Fig. 4). Furthermore, no trend toward increasing LC50 values was observed.

**Table 4:** Lethal concentrations (LC50) (based on percentage control) and resistance ratio (RR) of fluralaner for engorged female *Rhipicephalus microplus* ticks under laboratory conditions (27 ± 1 °C and RH 80 ± 5%) before and after implementation of different treatment protocols

Period/group	LC_50_ μg/mL	CI (95%)	Slope	RR	Status
Pre-treatment*	0.47	0.45 – 0.49	1.42	…	…
Group T01	0.46	0.44 – 0.47	2.89	0.98	Susceptible
Group T02	0.41	0.39 – 0.42	2.99	0.87	Susceptible

95% CI = 95% confidence interval

RR = resistance ratio

^*^Initial test performed before the division of the groups to carry out strategic control study

Group T01: animals treated with the formulation containing alphacypermethrin + chlorpyrifos + ethion (Potenty^®^). Tests performed after completion of the strategic control study

Group T02: animals treated with the formulation containing fluralaner (Exzolt 5%^®^). Tests performed after completion of the strategic control study

## Discussion

This study presents unprecedented information on the effect of implementing strategic tick control in cattle using fluralaner on the phenotypic susceptibility profile of a *R. microplus* population. In the pasture, the number of *R. microplus* larvae declined significantly from the second month of monitoring after the beginning of the strategic control with fluralaner, while in cattle, a reduction in tick infestation was observed since the first day of monitoring. The subpopulation exposed to the four fluralaner treatments remained susceptible to the acaricide, and no increase in LC50 values was observed.

In tropical regions with well-defined rainy and dry seasons, such as in the Midwest and Southeast of Brazil, strategic control of *R. microplus* often begins in the spring, at the start of the rainy season, which coincides with the first tick generation, when adult stages begin to be seen on animals [13, 29, 39]. This is because during the dry season, environmental conditions are unfavorable for tick development, which naturally reduces infestations in pastures and consequently on animals. With the onset of rainfall, environmental conditions favor the survival of ticks in the environment, and infestations begin to be observed on animals, but tick burdens in cattle and pastures are still low because of the previously observed unfavorable conditions [6, 14, 18, 25, 39, 40]. However, due to the specific conditions for conducting this study, the strategic control protocol was initiated in the summer, during the second generation [13, 29], to enable the collection of engorged females in sufficient numbers for perfom laboratory bioassays. As fluralaner is highly effective against *R. microplus* [10, 11], initiating the strategic control protocol between July and October would prevent the collection of sufficient quantities of engorged females required for laboratory bioassays.

Due to the previously mentioned circumstances, strategic control was not initiated during the ideal period (first generation). However, starting control in the second generation allowed evaluation of fluralaner under a higher parasitic challenge, unlike other studies that began in the first [13, 18] or second generation with lower parasite challenge [17]. These results provide valuable and unprecedented information on the effectiveness of fluralaner under high parasite challenge conditions. At the beginning of the study, cattle had a mean of 70 females on one side and a mean of 900 larvae per sampling area in each paddock. Regarding tick infestation in animals, the 7-day interval after the first fluralaner treatment was sufficient to eliminate 100% of the ticks from the cattle. This finding is consistent with the literature, which indicates that the 7-day interval after fluralaner application is generally sufficient to achieve 100% efficacy [10, 11, 17].

The cattle treated with fluralaner remained tick-free after the start of the study in 60.5% of observations (23/38), and even in periods with ticks, the count never exceeded a mean of 8.9 ticks per animal. However, in the control group, infestations were observed in all evaluations, with counts exceeding 50 ticks on one side in some evaluations (Additional file 1: Table S1). These results are similar to the data found in strategic control studies with fluralaner conducted in tropical and subtropical regions of Brazil (Midwest, Southeast, and South) with different cattle breeds, in which it was also observed that the animals were free of ticks in > 70% of the observations, and the count never exceeded the mean number of 14.6 ticks per animal [13, 17, 18]. These findings were expected, since fluralaner has high efficacy against R. microplus and only became commercially available in Brazil in the second half of 2022 [10]; it has only been on the market for 3 years, and there are still no records of resistant populations [11, 41].

Regarding pasture infestation, a significant reduction in the number of *R. microplus* larvae was observed in the paddock treated with fluralaner (T02) from the second assessment (day 60), indicating that approximately 2 months are required for this significant reduction to occur. This fact is probably related to the survival time of larvae in the pasture, which can vary from 10 to > 100 days, depending on favorable climatic conditions [25, 42–44]. Thus, in the 30-day assessment, the presence of larvae at high levels (mean > 800) in the group treated with fluralaner is due to oviposition by fully engorged females that detached from cattle before the first treatment. In the evaluation from day + 60, the reduction in larval infestation in the pasture is related to the elimination of larvae when they are intoxicated with fluralaner during blood feeding on treated hosts and mortality from starvation due to natural factors associated with the longevity of the larvae in the environment [25, 42].

In the paddock where the animals were treated with fluralaner, from day + 60 onwards, larval levels remained low in the pastures, with averages of < 50 larvae per paddock throughout the remainder of the study, while in the control group, treated with a spray formulation, counts ranged from 1000 to 12,000 larvae per paddock. This indirect effect, popularly known as “pasture cleaning”, obtained by treating animals is one of the main objectives of strategic control [14]. This has been described in protocols with fluralaner [13] and with combinations of synthetic pyrethroids and organophosphates in tropical regions [6]. In the present study, this “pasture cleaning” result is associated with three factors: (i) the high susceptibility of *R. microplus* populations to fluralaner [11], leading to intoxication of ticks during the parasitic phase; (ii) the long period of persistent efficacy of the molecule [10, 11]; (iii) the retreatment criterion based on the presence of females < 4 mm in length in at least 30% of the animals in the group [6], which reduces the probability of engorged females detaching before being exposed to fluralaner [13].

The evaluation of the phenotypic profile of the *R. microplus* population prior to the implementation of strategic control indicated susceptibility to fluralaner, with RR > 1.1 and 100% mortality in DD tests. Susceptibility prior to the implementation of strategic control was already expected for two reasons: (i) fluralaner was introduced 3 years ago in the Brazilian market [10]; (ii) on the property where the research was conducted, no treatment with fluralaner had been performed to date. These data are consistent with the first surveys of fluralaner susceptibility profiles conducted [11, 41, 45]. For example, Chagas et al. [23] evaluated the phenotypic susceptibility profile of 200 populations of *R. microplus* from all five regions of Brazil (21 federal units), and the results indicated that all populations were susceptible to fluralaner.

The evaluation of the phenotypic profile of the subpopulations (T01 and T02) of *R. microplus* after the implementation of strategic control for 9 months and the administration of four treatments indicated susceptibility to fluralaner (unfed larvae and engorged females), with RR < 1.2 and 100% mortality in the DDs. Although 9 months is a short period for the development of resistance, there is evidence of phenotypic changes at similar intervals for other acaricides. Sarli et al. [46] reported a significant increase in LC_50_ after three consecutive applications of long-acting ivermectin, characterizing incipient resistance. Subsequently, Sarli et al. [37] observed an increase in LC_50_ after three treatments with ivermectin and fipronil, either alone or in combination, although the transition from susceptible to resistant occurred only with fipronil. In contrast, Nicaretta et al. [6] found, in a 4-year study, that susceptibility to a combination of chlorpyrifos + high-cis cypermethrin was maintained, with a slight increase in the LC50 value in laboratory tests. In this sense, it would be interesting to conduct long-term studies with fluralaner, as carried out by Nicaretta et al. [6], or even in populations with a history of exposure to fluralaner. A curious aspect is that, in both unfed larvae and engorged females of *R. microplus*, lower LC_50_ values were observed in the subpopulation exposed to the four treatments with fluralaner. Possibly, the reduction in LC_50_ in the subpopulation exposed to fluralaner (T02) is related to natural variability within this subpopulation [11] or to spatiotemporal variability [16] in the susceptibility of this *R. microplus* subpopulation to fluralaner.

Finally, we emphasize that this study does not constitute a recommendation to initiate strategic control in the second generation or to continue the use of fluralaner-only treatments. Some authors suggest that alternating acaricides with different modes of action to control *R. microplus* may help preserve molecules and slow the selection of resistance [2, 8, 47–49]. However, there are still no studies in this regard that test this hypothesis for most commercially available acaricides, including fluralaner. In this context, more studies like this one are needed to better understand the development of resistance over longer periods and in populations with a history of exposure to fluralaner, across different regions and climatic conditions, among different cattle breeds, and under different fluralaner use patterns, including both fluralaner-only use and fluralaner alternating with other acaricides.

## Conclusions

The strategic control protocol with fluralaner in taurine cattle significantly reduced *R. microplus* burden in both animals and pastures, indicating that fluralaner may be a valuable tool for addressing scenarios of high parasitic challenge. When the control protocol was initiated during the second tick generation, the animals were already free of ticks after 7 days, while a significant reduction in larvae in the pasture was observed after 60 days. The subpopulation exposed to fluralaner remained susceptible to this isoxazoline after implementation of the strategic control protocol. This is the first study to perform this type of evaluation with fluralaner, including analyses using LIT (RR and DDs) and AIT (RR). Further studies in different regions and production systems, with longer experimental periods and on farms with a history of fluralaner use, are needed to generate robust evidence on effective strategies to monitor and slow the selection of acaricide-resistant ticks.

## Supplementary Information

Supplementary Material 1.

## Data Availability

All data are included as tables and figures in the article.
