# Supplementary material for: Susceptibility of Rhipicephalus microplus to fluralaner: assessment before and after implementation of a strategic control protocol in taurine cattle in a tropical region
Source: Parasit Vectors. 2026 Jun 16;19:363. doi: 10.1186/s13071-026-07491-1 (PMC13508281; doi:10.1186/s13071-026-07491-1)
Supplement: Supplementary file 1 [file 13071_2026_7491_MOESM1_ESM.docx]

**Supplementary information**

**Additional file 1: Table S1.** Mean count of female *Rhipicephalus microplus* ticks, measuring between 4.5 and 8 mm in length, on cattle treated with different formulations containing fluralaner (Exzolt 5%^®^, pour-on) or alphacypermethrin + chlorpyrifos + ethion (Potenty^®^, spray).

|  | Experimental groups | |  |
| --- | --- | --- | --- |
| Days | Group T01: alphacypermethrin + chlorpyrifos + ethion  (mean ± standard error) | Group T02: fluralaner  (mean ± standard error) | *P*  (Kruskal-Wallis) |
| 0^*,α,β^ | 71.1 ± 60.3 ^a^ | 69.9 ± 48.6 ^a^ | 0.718 |
| 7 | 7.6 ± 8.1 ^a^ | 0.0 ± 0.0 ^b^ | <0.001 |
| 14 | 14.2 ± 14.4 ^a^ | 0.0 ± 0.0 ^b^ | <0.001 |
| 21 | 8.0 ± 15.8 ^a^ | 0.0 ± 0.0 ^b^ | 0.002 |
| 28 ^α, β^ | 65.9 ± 56.3 ^a^ | 0.0 ± 0.0 ^b^ | <0.001 |
| 35 | 19.1 ± 13.6 ^a^ | 0.0 ± 0.0 ^b^ | <0.001 |
| 42 ^β^ | 8.7 ± 13.7 ^a^ | 6.9 ± 20.6 ^b^ | 0.009 |
| 49 | 2.6 ± 3.2 ^a^ | 0.0 ± 0.0 ^b^ | <0.001 |
| 56 ^α^ | 43.4 ± 33.4 ^a^ | 0.0 ± 0.0 ^b^ | <0.001 |
| 63 | 3.7 ± 5.3 ^a^ | 0.0 ± 0.0 ^b^ | <0.001 |
| 70 | 11.0 ± 9.3 ^a^ | 0.0 ± 0.0 ^b^ | <0.001 |
| 77 | 2.6 ± 5.3 ^a^ | 0.0 ± 0.0 ^b^ | <0.001 |
| 84 | 13.2 ± 12.4 ^a^ | 0.0 ± 0.0 ^b^ | <0.001 |
| 91 ^α, β^ | 33.3 ± 12.6 ^a^ | 0.3 ±0.9 ^b^ | <0.001 |
| 98 | 10.2 ± 2.0 ^a^ | 0.0 ± 0.0 ^b^ | <0.001 |
| 105 | 19.8 ± 36.2 ^a^ | 0.0 ± 0.0 ^b^ | <0.001 |
| 111 | 2.5 ± 1.3 ^a^ | 0.0 ± 0.0 ^b^ | <0.001 |
| 119 ^α^ | 47.3 ± 36.3 ^a^ | 0.0 ± 0.0 ^b^ | <0.001 |
| 126 | 2.7 ± 1.3 ^a^ | 0.0 ± 0.0 ^b^ | <0.001 |
| 133 ^β^ | 0.5 ± 0.7 ^a^ | 0.3 ± 0.7 ^a^ | 0.282 |
| 140 | 25.8 ± 32.6 ^a^ | 0.0 ± 0.0 ^b^ | <0.001 |
| 147 ^α^ | 43.3 ± 58.5 ^a^ | 0.0 ± 0.0 ^b^ | <0.001 |
| 154 | 13.3 ± 16.1 ^a^ | 0.0 ± 0.0 ^b^ | <0.001 |
| 161 | 13.7 ± 24.2 ^a^ | 0.0 ± 0.0 ^b^ | <0.001 |
| 168 | 8.1 ± 10.9 ^a^ | 0.0 ± 0.0 ^b^ | <0.001 |
| 175 | 22.3 ± 8.9 ^a^ | 0.0 ± 0.0 ^b^ | <0.001 |
| 182 ^α^ | 37.6 ± 28.0 ^a^ | 0.0 ± 0.0 ^b^ | <0.001 |
| 189 | 11.9 ± 20.7 ^a^ | 0.1 ± 0.4 ^b^ | <0.001 |
| 196 | 5.8 ± 4.4 ^a^ | 0.6 ± 1.2 ^b^ | <0.001 |
| 203 | 4.5 ± 15.2 ^a^ | 3.6 ± 10.1 ^a^ | 0.331 |
| 210 ^α^ | 55.4 ± 132.6 ^a^ | 0.1 ± 0.4 ^b^ | 0.005 |
| 217 | 3.3 ± 5.3 ^a^ | 0.1 ± 0.4 ^b^ | 0.029 |
| 224 | 1.4 ± 2.2 ^a^ | 0.3 ± 0.8 ^b^ | 0.023 |
| 231 | 1.2 ± 1.8 ^a^ | 0.4 ± 0.6 ^a^ | 0.276 |
| 238 | 2.1 ± 3.8 ^a^ | 0.1 ± 0.3 ^b^ | 0.014 |
| 245 | 6.7 ± 5.6 ^a^ | 0.1 ± 0.3 ^b^ | <0.001 |
| 252 | 12.4 ± 10.4 ^a^ | 0.1 ± 0.3 ^b^ | <0.001 |
| 259 | 15.3 ± 11.3 ^a^ | 1.8 ± 3.0 ^b^ | 0.001 |
| 266 | 23.3 ± 26.0 ^a^ | 8.9 ± 10.0 ^b^ | 0.041 |

*: corresponds to the average number of ticks counted on days -3, -2, and -1.

α: when animals from T01 were treated with the formulation containing alphacypermethrin + chlorpyrifos + ethion (Potenty^®^).

β: when animals from T02 were treated with the formulation containing fluralaner (Exzolt 5%^®^).

Averages followed by different letters on the same row indicate a significant difference (p < 0.05), observed using the Kruskal-Wallis test.
